# Chemical Structure and Immunomodulating Activities of an α-Glucan Purified from *Lobelia chinensis* Lour

**DOI:** 10.3390/molecules21060779

**Published:** 2016-06-15

**Authors:** Xiao-Jun Li, Wan-Rong Bao, Chung-Hang Leung, Dik-Lung Ma, Ge Zhang, Ai-Ping Lu, Shun-Chun Wang, Quan-Bin Han

**Affiliations:** 1 Institute of Chinese Materia Medica, Shanghai University of Traditional Chinese Medicine, Shanghai 201203, China; lixiaojun@hkbu.edu.hk; 2 School of Chinese Medicine, Hong Kong Baptist University, Hong Kong 999077, China; wanrong@hkbu.edu.hk (W.-R.B.); zhangge@hkbu.edu.hk (G.Z.); aipinglu@hkbu.edu.hk (A.-P.L.); 3 State Key Laboratory of Quality Research in Chinese Medicine, Institute of Chinese Medical Sciences, University of Macau, Macao 999078, China; duncanleung@umac.mo; 4 Department of Chemistry, Hong Kong Baptist University, Hong Kong 999077, China; edmondma@hkbu.edu.hk

**Keywords:** *Lobelia chinensis*, NMR, RAW 264.7, immunomodulating, TLR4

## Abstract

A neutral α-glucan, named BP1, with a molecular mass of approximately 9.45 kDa, was isolated from *Lobelia chinensis* by hot-water extraction, a Q-Sepharose Fast Flow column and Superdex-75 column chromatography. Its chemical structure was characterized by monosaccharide analysis, methylation analysis and analysis of its FT-IR, high performance gel permeation chromatography (HPGPC) and 1D/2D-NMR spectra data. The backbone of BP1 consists of →_6_α-d-Glcp^1^→_6,3_α-d-Glcp^1^→(_6_α-d-Glcp^1^)x-_6,3_α-d-Glcp^1^-(_6_α-d-Glcp^1^)y→. The side chains were terminal α-d-Glcp^1^→ and α-d-Glcp^1^→ (_6_α-d-Glcp^1^)z→_4_α-d-Glcp^1^→_3_α-d-Glcp^1^→_4_α-d-Glcp^1^→ (x + y + z = 5), which are attached to the backbone at O-3 of _3,6_α-d-Glcp^1^. The results of the effect of BP1 on mouse macrophage cell line RAW 264.7 indicate that BP1 enhances the cell proliferation, phagocytosis, nitric oxide production and cytokine secretion in a dose-dependent manner. Because the inhibitor of Toll-like receptor 4 blocks the BP1-induced secretion of TNF-α and IL-6, we hypothesize that α-glucan BP1 activates TLR4, which mediates the above-mentioned immunomodulating effects.

## 1. Introduction

*Lobelia chinensis*, commonly known as Chinese lobelia, Herba Lobellae Chinensis, aze mushiro and mizo kakushi, grows wild throughout East Asia. It is one of the most used anti-cancer herbs in Chinese Medicine [1]. Its chemical profile is very complex. The current phytochemical studies have found various types of chemical components, including piperidine alkaloids, coumarins, terpenoids and saponins [2,3]. These chemicals show various biological activities, including anti-bacterial, anti-venom, anticancer, anti-viral and anti-inflammatory activities [4,5,6,7,8]. In addition to these small molecules, this herb has a large amount of polysaccharides, the content of which can reach 25% by weight of the dry herb [9]. Little is known about the chemistry and bioactivity of the polysaccharides of this herb.

Plant polysaccharides have shown many bioactivities [10,11], whose ability to modulate immune function is popularly considered a major aspect. Many polysaccharides have been reported to be able to activate macrophages [12,13,14], and this activation plays an important role in the immune response. The present study aims to analyze the chemistry of polysaccharides purified from the water extract of *Lobelia chinensis* and to explore their immune modulating effects on the RAW 264.7 cell line. A neutral α-glucan named BP1, with a molecular mass of approximately 9.45 kDa is isolated from *Lobelia chinensis*, and its chemical structure was characterized by chemical and spectral analyses. BP1 was able to enhance the cell proliferation, phagocytosis, nitric oxide production and cytokine secretion in a dose-dependent manner.

## 2. Results and Discussion

### 2.1. Isolation and Purification of BP1

**Figure 1:**
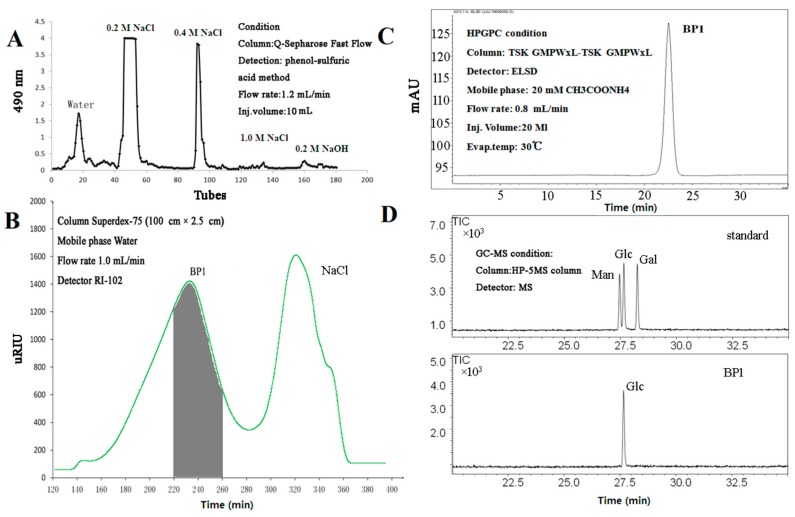
(**A**) The Q-Sepharose column chromatography of crude polysaccharide extracted from *Lobelia chinensis*; (**B**) gel filtration chromatography of the major fraction (0.2 NaCl elution) on Superdex 75; (**C**) homogeneity of BP1 as measured by high performance gel permeation chromatography (HPGPC); (**D**) monosaccharide composition of BP1 (upper: reference standards; lower: BP1).

Separation of the crude polysaccharide from *Lobelia chinensis* using fast-flow chromatography on Q-Sepharose generated five fractions: water, 0.2 M NaCl, 0.4 M NaCl, 1.0 M NaCl and 0.2 M NaOH fractions (Figure 1A). Further purification of the major fraction (0.2 M NaCl, 598 mg) using gel filtration chromatography on Superdex 75 (Figure 1B) yielded a purified polysaccharide, which was shown to be homogeneous in high performance gel permeation chromatography (HPGPC) (Figure 1C). We named this polysaccharide BP1. Its average molecular weight was determined to be approximately 9450 Da.

### 2.2. FT-IR Spectral Analysis

The FT-IR spectra of BP1 (Figure S1) were analyzed as follows [15]: a strong and broad peak at around 3367 cm^−1^ was attributed to the vibration of the O-H bond; a weak absorption peak at approximately 2932 cm^−1^ was attributed to the stretching vibration of C-H bond; these two signals characterized the absorption spectra of polysaccharides. Furthermore, the band at 1639 cm^−1^ can be attributed to the H-O-H bond, while the signals from 1453 cm^−1^ to 1332 cm^−1^ were attributable to –CH (O–CH_2_) flexural vibrations, according to [16,17]. The band at 1131 cm^−1^ ws due to the C-O-C bond and glycosidic bridge, and the band at 1200 cm^−1^ and 1020 cm^−1^ might be given by the C–O stretching vibrations. The band at 846 cm^−1^ was ascribed to α-configuration of the polysaccharide. Absorption at 920 cm^−1^ indicated the existence of pyranose sugars [18].

### 2.3. Protein Assay and Monosaccharide Composition Analysis

BP1 was determined to be protein-free as measured via the Quick Start Bradford Protein Assay. The monosaccharide composition analysis using GC-MS indicated that BP1 was composed of glucose (Figure 1D).

### 2.4. Methylation Analysis

BP1 was subjected to methylation analysis to determine the linkage types [19,20]. According to the methylated derivatives, as shown in Table 1, the sugar residues of BP1 were determined to be 2,3,4,6-Me4-Glcp, 2,4,6-Me3-Glcp, 2,3,6-Me3-Glcp, 2,3,4-Me3-Glcp, 2,3-Me2-Glcp, 2,4-Me2-Glcp and 2-Me1-Glcp, indicating the existence of t-, 1,6-, 1,4-, 1,3-, 1,3,6-Glc, respectively.

### 2.5. NMR and Structure Analysis

The ^1^H- and ^13^C-NMR spectra of BP1 were carefully assigned after an integrative consideration of the above-mentioned results, the 1D/2D-NMR spectra of BP1. The results are shown in Table 2.

**Figure 2:**
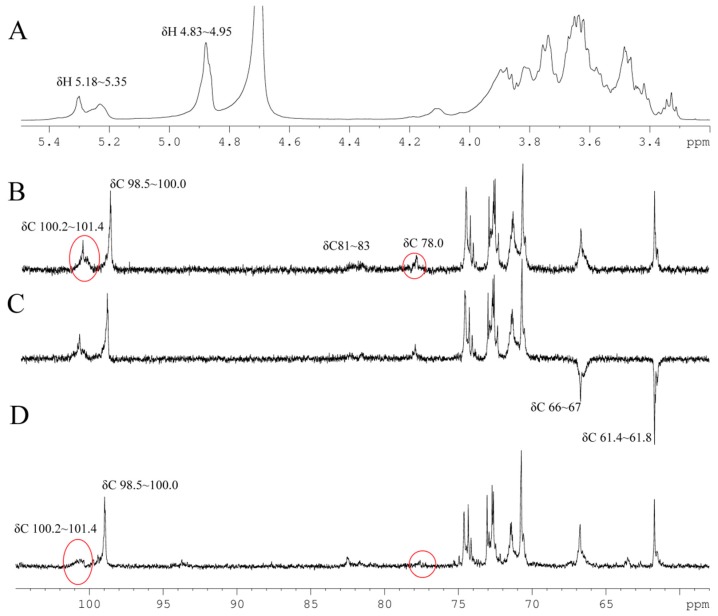
1D-NMR spectra of BP1. (**A**) ^1^H-NMR; (**B**) ^13^C-NMR; (**C**) DEPT135-NMR and (**D**) ^13^C-NMR of BP1a. The signals highlighted in red indicate the changes of C1, C3 of _3_α-d-Glcp^1^ and C4 of _4_α-d-Glcp^1^ caused by partial acid hydrolysis.

**Figure 3:**
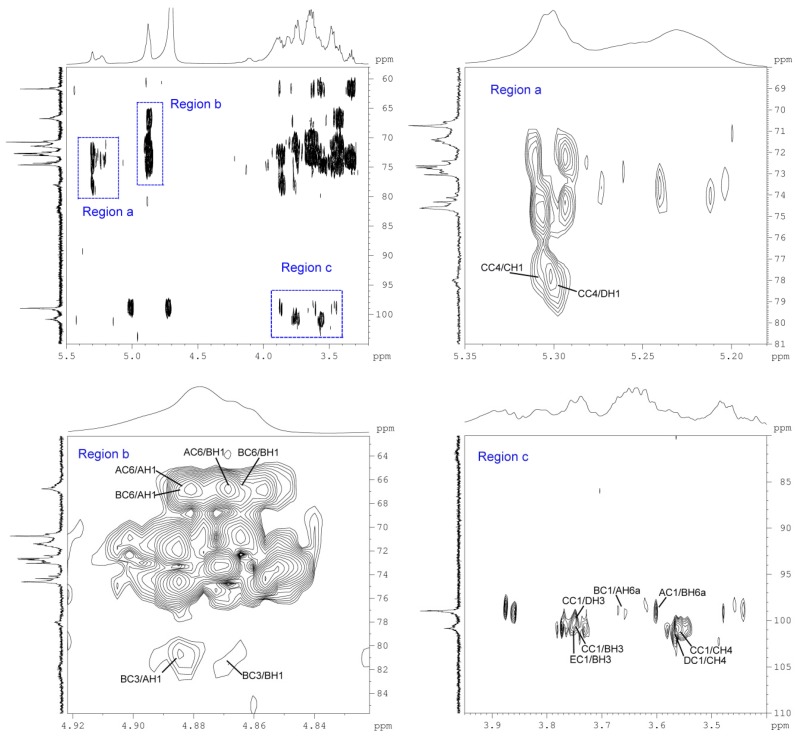
HMBC spectra of BP1: The inter-residue ^1^H-^13^C long-range correlations used for linkage and sequence assignments are listed in Table 3.

The anomeric signals were analyzed firstly. The proton signals around δ_H_ 4.83~4.95 ppm and δ_H_ 5.18~5.35 ppm in the ^1^H-NMR spectrum (Figure 2A) exhibited a peak area ratio of about 1.9:1; therefore the signals at δ_H_ 4.83~4.95 ppm were considered to represent the main linkages of BP1. The anomeric carbon signals at δ_C_ 98.5~100.0 ppm and δ_C_ 100.2~101.4 ppm from ^13^C-NMR (Figure 2B) showed a peak area ratio of about 1.76:1, so the signals at δ_C_ 98.5~100 ppm were attributed to the main anomeric carbon signals of BP1. It seems likely that the signals of δ_C_ 98.5~100.0/δ_H_ 4.83~4.95 were generated by 1,6-/1,3,6-linked Glc [21,22,24], which were some of the major linkage types according to methylation analysis (Table 1). This deduction was confirmed by the coupling of 98.5~100.0/4.83~4.95 in the HSQC spectrum (Figure 3B). The signals of δ_C_ 100.2~101.4/δ_H_ 5.18~5.35 were assigned to 1,3-/1,4-linked Glc and terminal Glc. In addition, compared to the published data of β*-*d-glucopyranose [20,25], the anomeric signals of BP1 were significantly shifted up field, which indicated that BP1 has an α-configuration [26]. This deduction was confirmed by the NOE interactions between H1 and H2 of residues 1,6-, 1,3,6- and 1,4-linked Glc.

In the DEPT-135 spectrum (Figure 2C), the signals at δ_C_ 66~67 ppm were most likely due to the C6 of 1,6-/1,3,6-linked Glc, and the signals at δ_C_ 61.4~61.8 ppm could be attributed to the C6 of 1,3-/1,4-linked Glc and terminal Glc. The signals at δ_C_ 78.0 may belong to the C4 of 1,4-linked Glc [23], while the two peaks at δ_C_ 81 and δ_C_ 83 could be attributed to the C3 of 1,3-linked Glc and 1,3,6-linked Glc, because they were significantly shifted up field compared to the chemical shift of carbon with a free hydroxyl [27,28].

The sequence of the monosaccharides in the sugar chain of BP1 was established by the analysis of the heteronuclear multiple bond correlation (HMBC) spectrum (Figure 3, Table 3). For residue _6_α-d-Glcp^1^, the anomeric proton showed a strong cross peak with the C6 of residue _3,6_α-d-Glcp^1^, and the anomeric carbon of residue _6_α-d-Glcp^1^ also had a strong coupling with H6b of residue _3,6_α-d-Glcp^1^, which confirmed the presence of _6_α-d-Glcp^1^→_6,3_α-d-Glcp^1^. The correlation between C1 of residue _3,6_α-d-Glcp^1^ and H6a of residue _6_α-d-Glcp^1^ and that between the anomeric proton of residue _3,6_α-d-Glcp^1^ with C6a of residue _6_α-d-Glcp^1^ further indicated the existence of _6,3_α-d-Glcp^1^→_6_α-d-Glcp^1^. These results suggest that the backbone of BP1 was composed of a _6,3_α-d-Glcp^1^ linkage and a _6_α-d-Glcp^1^ linkage.

Further, the interactions between C1 of residue _4_α-d-Glcp^1^ with H3 of residue _3,6_α-d-Glcp^1^ and between H1 of residue _4_α-d-Glcp^1^ with C3 of residue _3,6_α-d-Glcp^1^ indicated the presence of of _4_α-d-Glcp^1^→_3,6_α-d-Glcp^1^, suggesting that the side chain _4_α-d-Glcp^1^ was linked to the backbone at O-3 of the residue _3,6_α-d-Glcp^1^. In addition, C1 of residue _4_α-d-Glcp^1^ also exhibited a strong coupling with H3 of residue _3_α-d-Glcp^1^, which indicated the presence of _4_α-d-Glcp^1^→_3_α-d-Glcp^1^. The trans-glycosidic correlation between C1 of residue _3_α-d-Glcp^1^ and H4 of residue 4α-d-Glcp1, together with that between H1 of residue 3α-d-Glcp1 and C4 of residue _4_α-d-Glcp^1^ proved the presence of _3_α-d-Glcp^1^→_4_α-d-Glcp^1^. Since the ratio of the main residues _4_α-d-Glcp^1^:_3_α-d-Glcp^1^ is about 2:1, a fragment of →_4_α-d-Glcp^1^→_3_α-d-Glcp^1^→_4_α-d-Glcp^1^ may exist. Furthermore, the peak of _4_α-d-Glcp^1^→_3,6_α-d-Glcp^1^ was shown in HMBC, which allows one to infer that a side chain of →_4_α-d-Glcp^1^→_3_α-d-Glcp^1^→_4_α-d-Glcp^1^ might be connected to the backbone through the O-3 site of _3,6_α-d-Glcp^1^. Finally, the presence of α-d-Glcp^1^→_3,6_α-d-Glcp^1^ was deduced from the correlation between C1 of residue α-d-Glcp^1^ and H3 of residue _3,6_α-d-Glcp^1^. Another side chain was composed by α-d-Glcp^1^, which joined the backbone through the O-3 site of _3,6_α-d-Glcp^1^.

**Figure 4:**
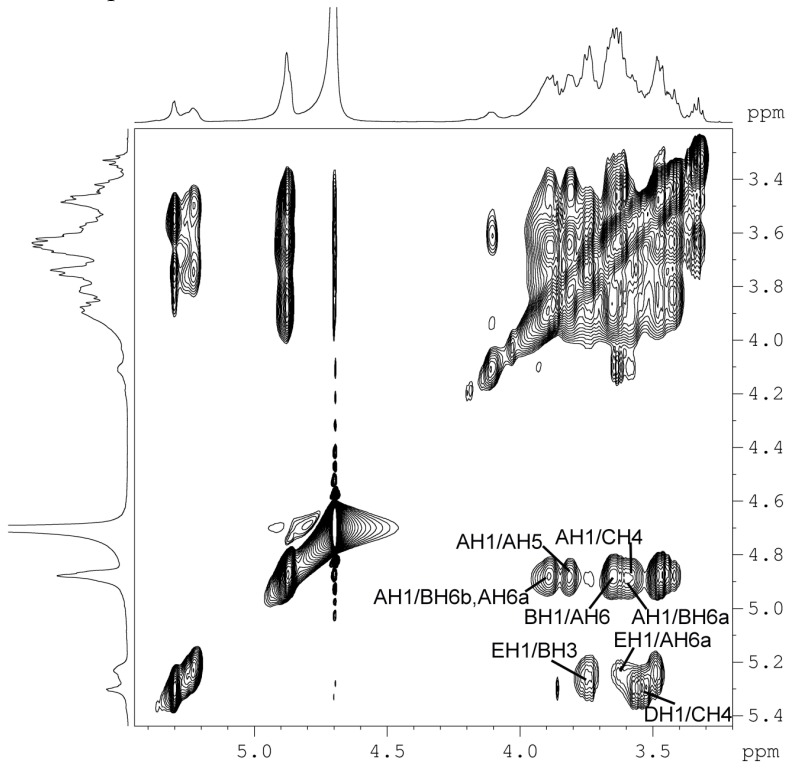
NOESY spectrum of BP1. NOE correlations are labelled with residue and proton/carbon numbers, as listed in Table 4.

More evidence regarding the precise structure of the sugar chain of BP1 was found in the NOESY (nuclear Overhauser effect spectroscopy) (Figure 4, Table 4). The NOEs between AH1/BH6ab, DH1/CH4 and EH1/BH3 confirmed the deductions of _6_α-d-Glcp^1^→_6__,3_α-d-Glcp^1^, _3_α-d-Glcp^1^→_4_α-d-Glcp^1^, and α-d-Glcp^1^→_3__,6_α-d-Glcp^1^, and NOEs between EH1/AH6ab and AH1/CH4 further confirmed the linkage of α-d-Glcp^1^→_6_α-d-Glcp^1^, and _6_α-d-Glcp^1^→_4_α-d-Glcp^1^. Based on the above evidence, the sequence of the residues of α-d-Glcp^1^→_6_α-d-Glcp^1^→_4_α-d-Glcp^1^→ was proposed.

Taking all the NOE and HMBC evidence together, a side chain of α-d-Glcp^1^→_6_α-d-Glcp^1^→_4_α-d-Glcp^1^→_3_α-d-Glcp^1^→_4_α-d-Glcp^1^→ could be proposed, which was confirmed by the analysis of the partial acid hydrolysis of BP1. The anomeric signals at δ_C_ 100.2~101.4 and 98.5~100.0 ppm showed a peak area ratio of about 1:1.76 in the ^13^C-NMR spectrum of BP1 (Figure 2B), and the ratio significantly decreased to 1:3.7 after partial acid hydrolysis (Figure 2D). These results suggest that _4_α-d-Glcp^1^ and _3_α-d-Glcp^1^ could be cut off from the main chain by partial acid hydrolysis, indicating that they might exist as side chains. This deduction was confirmed by the decreased signal around δ_C_ 78.0 ppm due to C4 of _4_α-d-Glcp^1^.

**Figure 5:**
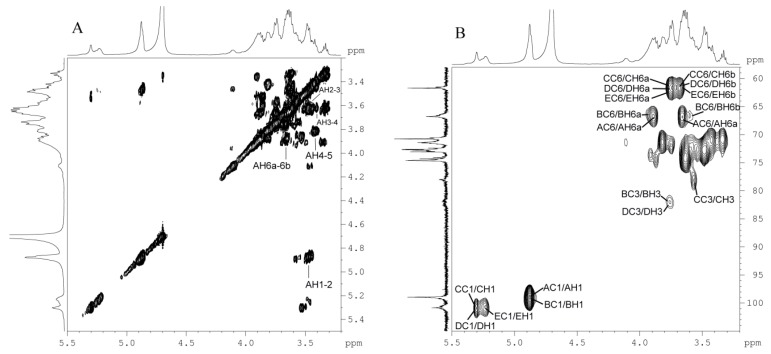
2D-NMR spectrum of BP1. (**A**) 1H-1H COSY spectra of BP1; (**B**) HSQC spectra of BP1. The cross-peaks are labelled with the residue and number; residue names are the same as those in Table 2.

Finally, with the aid of the ^1^H-^1^H COSY spectrum (Figure 5A) and the HSQC spectrum (Figure 5B), the ^1^D-NMR data were successfully assigned, and the results are presented in Table 2. The methylation analysis indicated that BP1 mainly consists of residues _6_α-d-Glcp^1^, _3,6_α-d-Glcp^1^, _4_α-d-Glcp^1^, _3_α-d-Glcp^1^ and α-d-Glcp^1^. Based on methylation analysis, we concluded that the ratio of the main residues _6_α-d-Glcp^1^:_3,6_α-d-Glcp^1^:_4_α-d-Glcp^1^:_3_α-d-Glcp^1^ is about 6:2:2:1. Therefore, we may infer that the backbone consists of residues following this order: →_6_α-d-Glcp^1^→_6,3_α-d-Glcp^1^→_6_α-d-Glcp^1^-_6,3_α-d-Glcp^1^-_6_α-d-Glcp^1^→. A side chain is attached to the backbone through O-3 of residue B. There are two branches that were α-d-Glcp^1^→_6_α-d-Glcp^1^→_4_α-d-Glcp^1^→_3_α-d-Glcp^1^→_4_α-d-Glcp^1^→ and terminal α-d-Glcp^1^→, which were located at the O-3 site of residue B (_3,6_α-d-Glcp^1^).

**Figure 6:**
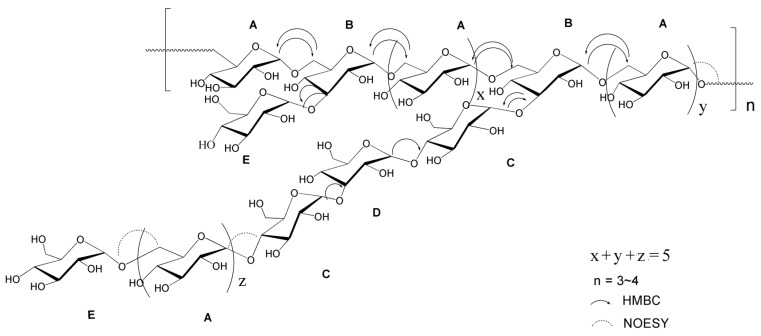
The proposed chemical structure of BP1. (**A**) _6_α-d-Glcp^1^; (**B**) _3,6_α-d-Glcp^1^; (**C**) _4_α-d-Glcp^1^; (**D**) _3_α-d-Glcp^1^; (**E**) α-d-Glcp^1^. The symbols of x, y and z were used to express the number of _6_α-d-Glcp^1^.

In summary, we conclude that the backbone consists of →_6_α-d-Glcp^1^→_6,3_α-d-Glcp^1^→(_6_α-d-Glcp^1^)x-_6,3_α-d-Glcp^1^-(_6_α-d-Glcp^1^)y→. The side chains were α-d-Glcp^1^→(_6_α-d-Glcp^1^)z→_4_α-d-Glcp^1^→_3_α-d-Glcp^1^→_4_α-d-Glcp^1^→ and α-d-Glcp^1^→, being attached to the backbone at O-3 of _3,6_α-d-Glcp^1^. According to the linkage ratio, as revealed by the methylation analysis, the total sum of x, y and z should be five. Additionally, the molecular weight of BP1 suggested that n should be 3–4. The proposed structure is shown in Figure 6. A literature research indicated that the α-glucan BP1 is the first purified polysaccharide obtained and identified from *Lobelia*, even though this genus includes more than 415 species [29].

### 2.6. MTT Assay

**Figure 7:**
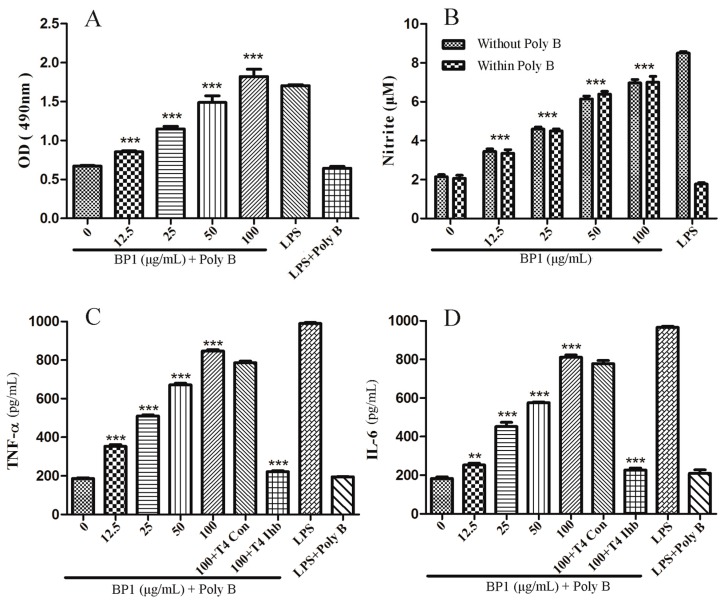
Immunomodulating effects of BP1. (**A**) Dose-dependent induction of the proliferation of RAW 264.7 cells; (**B**) dose-dependent effect on the production of NO by RAW 264.7 cells *in vitro*; (**C**) induction of RAW 264.7 cell TNF-α secretion *in vitro*, and the role of TLR4 in this bioactivity; (**D**) Induction of RAW 264.7 cell IL-6 secretion *in vitro*, and the role of TLR4 in this bioactivity. LPS (2 µg/mL) and LPS + polymyxin B (PolyB) served as the positive control and endotoxin-excluded control, respectively. Each value is expressed as the mean ± SD (*n* = 3). Level of significance: ** *p* < 0.01; *** *p* < 0.001 compared to untreated cells (at 0 µg/mL).

The results of the MTT assay of BP1’s effect on the cell proliferation of RAW 264.7 cells are shown in Figure 7A. The positive control LPS induced cell proliferation, and polymyxin B (PolyB) stopped it. Like LPS, BP1 showed significant induction of the proliferation of RAW 264.7 cells, in a dose-dependent manner at concentrations of 12.5, 25, 50, 100 µg/mL, when administered together with PolyB in order to suppress the effect of LPS. The results suggest that LPS’s impact could be excluded in the case of BP1.

### 2.7. Effect of BP1 on NO Release by RAW 264.7 Cells

In this study, the release of NO by RAW 264.7 cells after incubation with different concentrations of BP1 was determined (Figure 7B). As happens with LPS, BP1 also induced a strong NO release in a dose-dependent manner within the varied concentrations of 12.5, 25, 50, 100 µg/mL. PolyB inhibited the effect of LPS, but did not affect that of BP1.

### 2.8. Cytokine Production and the Role of TLR 4

Similar results were obtained in the determination of the effects of LPS and BP1 on the secretion of TNF-α and IL-6 by RAW 264.7 cells. LPS and LPS plus PolyB served as positive and endotoxin-excluded controls, respectively. The levels of TNF-α and IL-6 increased after LPS treatment and after BP1 + PolyB treatment (Figure 7C,D). When administrated with PolyB in order to suppress the effect of LPS, BP1 induced dose-dependent increases over the concentration range of 12.5, 25, 50, 100 μg/mL. Furthermore, when Toll-like receptor TLR 4 inhibitor (T4 Ihb) was added to the high-dose BP1 group (100 µg/mL), the production of both TNF-α and IL-6 was significantly suppressed, but the TLR 4 control (100 + T4 Con) did not show such significant difference. These results suggested that TLR 4 may play an important role in the signal transduction.

### 2.9. Assay of Phagocytosis

**Figure 8:**
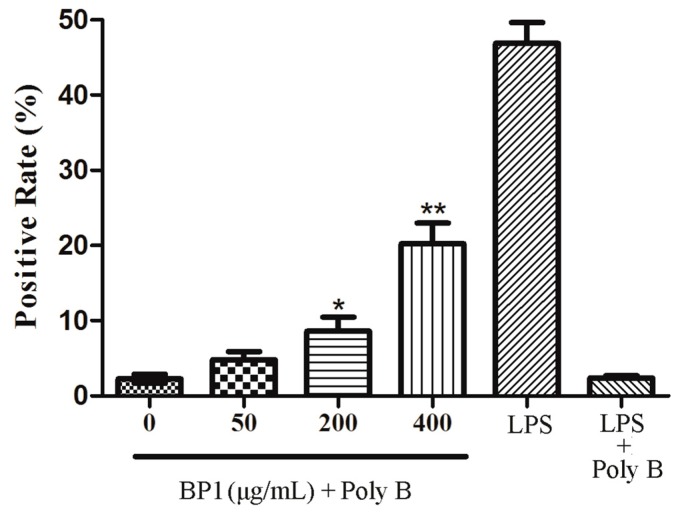
Inducing the effect of BP1 on the phagocytosis to FITC-labeled dextran (12 kDa) by RAW 264.7 cells in a dose-dependent manner. LPS (2 µg/mL) and LPS + polymyxin B served as the positive control and endotoxin-excluded control, respectively. Each value is expressed as the mean ± SD (*n* = 3). Level of significance: * *p* < 0.05, ** *p* < 0.01 compared to untreated cells (at 0 µg/mL).

The effect of BP1 on the phagocytosis to FITC-dextran by RAW 264.7 cells is shown in Figure 8. LPS and LPS plus PolyB served as positive and endotoxin-excluded controls, respectively. LPS at 2 µg/mL significantly induced the phagocytosis, and PolyB stopped it. BP1 at concentrations of 200 and 400 μg/mL, when administrated with PolyB in order to suppress the impact of LPS, induced the phagocytosis to FITC-dextran by RAW 264.7 cells.

Generally speaking, the bioactivities of polysaccharides are closely dependent on their structures, including monosaccharide composition and sequence, glucosidic bonds, configuration and chain conformation [30,31,32]. However, it has not been clear if the pattern-recognition receptors of the cell membrane might be specific to the α,β-configuration of polysaccharides. It has been known that Dectin-1 is the receptor responsible for the recognition of both β-(1,3) glucans [33] and α-(1,3) glucans [34]. TLR2 is the receptor responsible for the recognition of both β-(1,3) glucans [35] and α-(1,4) glucans [36]. TLR4 is the receptor responsible for the recognition of α-(1,4,6)-glucan [37]. In this study, TLR4 was found to be a potential receptor for the α-glucan BP1. Because it is not known whether TLR4 functions as a receptor for β-glucan, we cannot say whether TLR4 is configuration-specific. Therefore, some more research is still needed to determine the role of TLR4 in the immuno-modulating effects of β-glucans.

## 3. Materials and Methods

### 3.1. Materials

Samples of Herba Lobellae Chinensis, *i.e.*, dried *Lobelia chinensis*, were provided by the Kang Qiao Traditional Chinese Medicine Decoction Pieces Co. Ltd. (Shanghai, China), China, and were identified by Prof. Zhili Zhao (Shanghai University of Traditional Chinese Medicine, Shanghai, China 131120). Monosaccharide standards were all from Fluka, USA. Dextran standards and trifluoroacetic acid (TFA) and dimethyl sulfoxide (DMSO) were from Sigma-Aldrich (St. Louis, MO, USA). Other reagents were analytical grade and obtained from China Chemical Reagent Industry (Shanghai, China), unless otherwise specified.

### 3.2. Isolation and Purification of BP1

The dried herbs of *L. chinensis*, samples of approximately 1.0 kg, were weighed accurately, crushed and then refluxed in absolute ethanol at 80 °C for 2 h. The residues were extracted twice with 5 L of distilled water at 100 °C for 3 h. After centrifuging at 4000 rpm for 10 min, the supernatant was collected and condensed to 100 mL by rotary evaporation at 60 °C. The concentrated solution was precipitated overnight with four volumes of 95% EtOH in a refrigerator (4 °C). After centrifuging at 4000 rpm for 10 min, the precipitate was collected and freeze-dried, yielding crude polysaccharides (126 g).

A part of the crude polysaccharide (10 g) was dissolved in distilled water (50 mL) in a water bath at 60 °C and the solution was centrifuged at 12,000 rpm for 10 min. The above operation was repeated again. The supernatant (10 mL) was loaded onto a Q-Sepharose Fast Flow column (50 cm × 5 cm), which was eluted stepwise with distilled water, followed by 0.2 M and 0.4 M NaCl solution and the eluate was monitored using the phenol-sulfuric acid method [38,39,40]. The major fraction (the part of 0.2 M NaCl) was dialyzed with flowing tap-water and freeze-dried using a lyophilizer (Labconco, Kansas City, MO, USA). This fraction was further separated using a Superdex-75 column (100 cm × 2.5 cm), eluted with 0.2 M NaCl solution and monitored using an RI-102 refractive index detector (Lihui Biological Technology Co., Ltd., Suzhou, China). The fractions obtained (220–260 min) were combined, concentrated, dialyzed against flowing tap-water and lyophilized to obtain the major fraction BP1 (1.25 g).

### 3.3. Determination of the Molecular Weight

The molecular weight of the BP1 was estimated through high performance gel permeation chromatography (HPGPC) (Tosoh Bioscience, King of Prussia, PA, USA) by using combined columns of Shodex KS-802 and KS-804, which were eluted with a mobile phase of 0.2 M NaCl at a flow rate of 0.8 mL/min. The eluate was monitored by RI detector (RI-101 refractive index detector). To estimate the molecular weight, Shodex-packed columns were calibrated using a standard P-series of dextrans (P-5, P-10, P-20, P-50, P-100, P-200, P-400 and P-800). The molecular weight was calculated according to the method of Fan *et al.* and Liu *et al.* [41,42].

### 3.4. Determination of the Protein Content

The protein contents of the polysaccharide samples were determined using Quick start Bradford 1× Dye Reagent (Bio-Rad, Hong Kong, China). A standard curve was prepared with bovine serum albumin (BSA, 0–2.0 mg/mL). BP1 polysaccharide was dissolved in deionized water at a concentration of 2.0 mg/mL. The sample was mixed with diluted dye reagent and incubated at room temperature for 5 min. Then, the absorbance was measured at 595 nm using a microtiter plate reader.

### 3.5. Monosaccharide Composition Analysis

BP1 (2 mg) was hydrolyzed in 2 M TFA (2 mL) at 120 °C for 2 h in a sealed glass tube. The acid was removed under reduced pressure through repeated evaporation with MeOH. The hydrolysate was successively reduced with sodium borohydride, acetylated with acid anhydride (Ac_2_O) at 105 °C for 1 h, and the resulting alditol acetates were examined via GC-MS.

The gas chromatography-mass spectrometry (GC-MS) was performed on a Shimadzu QP-2010 instrument equipped with a Shimadzu AOC-20i auto sampler system and interfaced with a Shimadzu QP 2010S mass spectrometer (Shimadzu Corporation, Tokyo, Japan). Samples were injected in splitless mode on a DB-5 MS analytical column (30 m × 0.25-mm ID with a film thickness of 0.25-μm J & W Scientific, Folsom, CA, USA). Helium (purity, 99.999%) was used as a carrier gas at a constant flow rate of 1 mL/min. The gas chromatograph was operated in split mode with the split/splitless injector at the ratio of 1:5. The injector temperature was set at 250 °C. The temperature program was set at 140 °C, maintained for 5 min and increased to 250 °C for 5 min at an increment of 3 °C/min. The mass spectra were taken in SCAN mode at 300 eV from *m*/*z* 40 to 400, and the scan speed was 769 while the detector delayed for 5 min. The MS progress started at 5 min and ended at 44 min. Standard monosaccharides were carried out following the same procedure.

### 3.6. Methylation Analysis

Methylation analysis was carried out according to the modified Hakomori method [43]. Briefly, BP1 (10 mg) was accurately weighed and dissolved in 1.0 mL of DMSO, to which 1.0 mL of dimsyl sodium (SMSM, 0.25 g/mL) solution was added under water-free conditions. After incubation under stirring for 12 h at room temperature, 1 mL of iodomethane was added slowly into the above polysaccharide solution; the solution was kept under incubation in the dark for 5 h at room temperature. After that, it was dialyzed against flowing tap-water in a dialysis tube (with a molecular size cutoff at 1000 Da) for 24 h. The methylated polysaccharide was extracted three times with chloroform (2 mL each time); the chloroform extract was combined and dried using a reduced-pressure rotary evaporator. The above procedure was repeated three times, and the completeness of methylation was confirmed when the detection of the hydroxyl absorption in the IR spectrum failed. The methylated polysaccharide was hydrolyzed within 2 M TFA (2 mL); then, the generated methylated alditol acetates were analyzed by GC-MS. The temperature programming condition of GC was as follows: the column temperature of the column was initially 140 °C and was increased to 180 °C at 2 °C/min, then to 190 °C at 1 °C/min and to 280 °C at 10 °C/min and held for 5 min. The gas chromatograph was operated in split mode with the split/splitless injector at the ratio of 1:5. The injector temperature was set at 250 °C. The MS progress started at 5 min and ended at 44 min. Standard monosaccharide were carried out following the same procedure.

### 3.7. Partial Acid Hydrolysis

BP1 (50 mg) was dissolved in 5 mL of TFA (0.1 M) at 60 °C for 1 h. The hydrolysate solution was evaporated under reduced pressure, and MeOH was added into the residue to remove TFA. The product was lyophilized and dissolved in ultra-pure water. The degraded polysaccharide was isolated from the hydrolysis products by ethanol precipitation and was named BP1a, which was examined using HPGPC before the NMR test [44].

### 3.8. NMR Analysis

The ^1^H-NMR (400 MHz), ^13^C-NMR (100 MHz), 2D-NMR, including ^1^H-^1^H correlation spectroscopy (COSY), heteronuclear single-quantum coherence (HSQC) and heteronuclear multiple bond correlation (HMBC) spectra of BP1, were obtained on a Bruker Avance 400 spectrometer (Bruker BioSpin, Rheinstetten, Germany). BP1 (40 mg) was dissolved in 1 mL of D_2_O (99.9% D) and freeze-dried to complete deuterium exchange. The treated sample was dissolved again in 400 μL of D_2_O (99.9% D) to make the sample solution. Acetone was used as the internal standard (δ_H_ 2.225 ppm, δ_C_ 31.45 ppm). The Bruker TopSpin program was used to acquire and process the NMR data.

### 3.9. MTT Assay

The effect of BP1 on the proliferation of RAW 264.7 cells was measured using the MTT (3-[4,5-dimethylthiazol]-2,5-diphenyltetrazolium bromide, Sigma-Aldrich stain assay. Cells (1 × 10^4^ cells/well) were seeded in 96-well plates (Corning Inc., Corning, New York, NY, USA) and cultured overnight; they were then incubated with different concentrations of BP1 plus polymyxin B (PolyB 10 μg/mL, used to suppress the effect of LPS) for 24 h. LPS (2 μg/mL) and LPS plus PolyB (10 μg/mL) were used as controls. Following incubation, 30 µL of MTT solution (5 mg/mL in PBS) were added to each well, and the plates were incubated at 37 °C for another 4 h. Then, the medium was discarded, and 150 µL of dimethyl sulfoxide was added to dissolve the formazan crystals. The absorbance of each sample was read at 490 nm using a microplate reader (Thermo, Waltham, MA, USA).

### 3.10. Nitrite Assay

RAW 264.7 macrophages were seeded on a 24-well plate at the initial concentration of 2 × 10^5^ cells/well overnight. The cells were treated with 12.5, 25, 50 and 100 µg/mL of BP1 for 24 h. PolyB was added to the cells in order to monitor the impact of LPS. LPS (2 μg/mL) and LPS plus PolyB were used as controls. The production of NO was determined by assaying the accumulation of nitrite, in the culture medium having Griess reagent (Sigma-Aldrich). The nitrite concentration was calculated by comparison with standard sodium nitrite solutions by spectrophotometry, at 540 nm.

### 3.11. Cytokine Determination and the Effect of Toll-Like Receptor 4 Inhibitor

The production of tumor necrosis factor-alpha (TNF-α) and interleukin -6 (IL-6) was measured by using an ELISA kit (eBioscience, San Diego, CA, USA). Briefly, RAW 264.7 macrophages were seeded overnight on a 24-well plate at the initial concentration of 2 × 10^5^ cells/well. In the groups treated with TLR 4 inhibitor or TLR 4 inhibitor control (Novus Biologicals, Littleton, CO, USA), the cells were pretreated with TLR 4 inhibitor or its control and incubated at 37 °C, in an atmosphere of 5% CO_2_. One hour later, PolyB plus different concentrations of BP1 were added to the medium (system), and the cells were incubated for another 24 h. LPS (2 μg/mL) and LPS plus PolyB were used as controls. Cell-free supernatants were collected for the ELISA assay.

### 3.12. Phagocytic Assay

Fluorescein isothiocyanate-conjugated dextran (FITC-dextran, Mw 12,000) was prepared for the phagocytic assay according to a published study [45]. RAW 264.7 macrophages (4 × 10^5^ cells/well) were seeded in 12-well plates overnight and were then exposed to the various concentrations of 12.5, 25, 50 and 100 µg/mL of BP1 for 24 h, in which PolyB (10 μg/mL) was added to all of the cells in order to suppress the impact of LPS. LPS (2 μg/mL) and LPS plus PolyB (10 μg/mL) were tested as controls. After that, all of the media were removed, and the cells were treated with FITC-dextran (0.1 mg/mL), which was dissolved in the cell culture medium and incubated for 1 h at 37 °C in an atmosphere of 5% CO_2_. After incubation, cells were washed four times in cold phosphate-buffered saline (PBS) and were analyzed by flow cytometry (BD Biosciences, San Jose, CA, USA).

### 3.13. Data Analysis

All of the results were expressed as the mean ± SD/SEM. Statistical analysis was performed using one-way ANOVA. Data analysis was performed using GraphPad PRISM software Version 5.0 (GraphPad Software, San Diego, CA, USA). A value of *p* < 0.05 was chosen as the criterion of statistical significance.

## 4. Conclusions

A neutral α-glucan, named BP1, with a molecular mass of approximately 9.45 kDa was isolated from *Lobelia chinensis*. Its backbone mainly consists a _6_α-d-Glcp^1^ linkage. The side chains are terminal α-d-Glcp^1^→ and α-d-Glcp^1^→(_6_α-d-Glcp^1^)_z_→_4_α-d-Glcp^1^→_3_α-d-Glcp^1^→_4_α-d-Glcp^1^→, which are attached to the backbone at O-3 of _3,6_α-d-Glcp^1^. BP1 is able to enhance the cell proliferation, phagocytosis, nitric oxide production and cytokine secretion of RAW 264.7 cells in a dose-dependent manner. These effects might be mediated via TLR4, as the inhibitor of TLR4 is able to block the BP1-induced secretion of TNF-α and IL-6.

## Figures and Tables

**Table 1:** Linkages and individual molar ratios of BP1 elucidated by methylation analysis.

Methylation Sugars	Linkages	Retention Time (min)	Molar Ratio	Main Mass Fragment (*m*/*z* *, Intensity %)
2,3,4,6-Me4-Glcp	Glc-1	28.358	2.01	43 (100), 71 (20), 87 (25), 101 (65), 117 (50), 129 (48), 145 (40), 161 (45), 205 (10)
2,3,4-Me3-Glcp	Glc-1,6	36.061	5.97	43 (100), 71 (15), 87 (33), 99 (40), 101 (52), 117 (55), 129 (37), 161 (15), 173 (5), 189 (10), 233 (10)
2,3,6-Me3-Glcp	Glc-1,4	34.539	2.03	43 (100), 71 (5), 87 (18), 99 (15), 101 (20), 113 (15), 117 (45), 129 (5), 161 (3), 233 (20)
2,4,6-Me3-Glcp	Glc-1,3	33.862	0.99	43 (100), 71 (16), 87 (25), 99 (15), 101 (38), 113 (5), 117 (85), 129 (70), 161 (30), 173 (5), 233 (10)
2,4-Me2-Glcp	Glc-1,3,6	42.231	2.01	43 (100), 71 (5), 87 (25), 99 (10), 101 (5), 117 (32), 129 (45), 189 (15), 233 (5)

* The symbol *m* stands for mass and *z* stands for the charge number of ions. *m*/*z* represents mass divided by charge number.

**Table 2:** Signal assignments of the ^1^H- and ^13^C-NMR spectra of BP1 based on the analysis of H-HCOSY, HSQC and HMBC spectra [21,22,23,24].

Glycosyl Residues	C1	C2	C3	C4	C5	C6	
	H1	H2	H3	H4	H5	H6a	H6b
A: _6_α-d-Glcp^1^	99.0	73.1	74.3	70.8	71.4	66.8	
	4.85~4.90	3.44~3.48	3.60~3.64	3.40~3.43	3.80~3.83	3.64~3.66	3.86~3.90
B: _3,6_α-d-Glcp^1^	98.9	72.4	81.7	74.1	70.6	66.6	
	4.88~4.91	3.54~3.59	3.73~3.77	3.89~3.93	3.33~3.37	3.58~3.67	3.86~3.90
C: _4_α-d-Glcp^1^	101.0	71.4	74.6	78.0	71.6	61.8	
	5.29~5.32	3.52~3.54	3.84~3.88	3.55~3.58	3.73~3.77	3.67~3.69	3.73~3.75
D: _3_α-d-Glcp^1^	100.8	72.4	82.3	71.4	74.3	61.8	
	5.28~5.30	3.49~3.50	3.73~3.77	3.36~3.38	3.62~3.66	3.67~3.69	3.73~3.75
E: α-d-Glcp^1^	100.6	72.7	71.4	73.9	71.5	61.6	
	5.22~5.26	3.44~3.48	4.09~4.12	3.89~3.92	3.34~3.38	3.67~3.69	3.73~3.75

**Table 3:** The significant ^3^*J*_H,C_ correlations observed in the HMBC spectrum of BP1.

No.	Glycosyl Residues	Atom	Residue	δ_C_	Atom	Residue	δ_H_
A	_6_α-d-Glcp^1^	AH1	AC3 ^a^	74.6	AC1	AH2	3.44~3.48
	4.88	AH1	AC5 ^a^	71.4	AC1	AH6a	3.64~3.66
		AH1	AC6	66.8	AC1	BH6a ^b^	3.56~3.61
		AH1	BC3	81.7	AC1	CC3	3.84~3.88
		AH1	BC6 ^b^	66.6			
		AH1	CC2	71.4			
		AH1	CC3	74.6			
B	_3,6_α-d-Glcp^1^	BH1	AC2	73.1	BC1	AH6a ^c^	3.64~3.66
	4.87	BH1	AC4	70.8	BC1	BH6a	3.56~3.61
		BH1	AC6 ^c^	66.8			
		BH1	BC3 ^a^	81.7			
		BH1	BC5 ^a^	70.6			
		BH1	BC6	66.6			
C	_4_α-d-Glcp^1^	CH1	BC2	72.4	CC1	DH3 ^d^	3.73~3.77
	5.31	CH1	BC3 ^e^	74.6	CC1	BH3 ^e^	3.73~3.77
		CH1	DC2	72.4	CC1	CH4	3.57~3.58
		CH1	CC4	78.0			
D	_3_α-d-Glcp^1^	DH1	BC2	72.4	DC1	CH4 ^f^	3.57~3.58
	5.29	DH1	CC3	74.6			
		DH1	DC2	72.4			
		DH1	CC4 ^f^	78.0			
E	α-d-Glcp^1^	EH1	BC4	74.1	EC1	BH3 ^g^	3.73~3.77

^a^ For the presence of α-configurations; ^b^ For →_6_α-d-Glcp^1^→_6,3_α-d-Glcp^1^→; ^c^ For →_6,3_α-d-Glcp^1^→_6_α-d-Glcp^1^; ^d^ For →_4_α-d-Glcp^1^→_3_α-d-Glcp^1^→; ^e^ For →_4_α-d-Glcp^1^→_3,6_α-d-Glcp^1^→; ^f^ For →_3_α-d-Glcp^1^→_4_α-d-Glcp^1^→; ^g^ For α-d-Glcp^1^→_3,6_α-d-Glcp^1^→.

**Table 4:** Assignment of the NOESY spectra of BP1.

Anomeric Proton	NOE Contact Proton	δH	Glycosyl Residue
AH1	AH2	3.44~3.48	
	AH5	3.80~3.83	
	AH6a	3.64~3.66	
	AH6b	3.88~3.90	
	BH6a	3.59~3.61	→_6_α-d-Glcp^1^→_6,3_α-d-Glcp^1^→
	BH6b	3.88~3.90	→_6_α-d-Glcp^1^→_6,3_α-d-Glcp^1^→
	CH4	3.57~3.58	→_6_α-d-Glcp^1^→_4_α-d-Glcp^1^→
BH1	BH2	3.54~3.59	
	BH3	3.73~3.77	
CH1	CH2	3.52~3.54	
	CH3	3.73~3.77	
	BH3	3.73~3.77	
DH1	CH4	3.57~3.58	→_3_α-d-Glcp^1^→_4_α-d-Glcp^1^→
DH1	CH3	3.84~3.88	
EH1	BH3	3.73~3.77	α-d-Glcp^1^→_3,6_α-d-Glcp^1^→
	AH2	3.44~3.48	
	AH3	3.60~3.64	
	AH6a	3.64~3.66	α-d-Glcp^1^→_6_α-d-Glcp^1^→
	AH6b	3.88~3.90	α-d-Glcp^1^→_6_α-d-Glcp^1^→

## Supplementary Materials

Supplementary materials can be accessed at: http://www.mdpi.com/1420-3049/21/6/779/s1.
