# Supplementary material for: Chemical Structure and Immunomodulating Activities of an α-Glucan Purified from Lobelia chinensis Lour
Source: Molecules. 2016 Jun 15;21(6):779. doi: 10.3390/molecules21060779 (PMC6274272; doi:10.3390/molecules21060779)
Supplement: Supplementary file 1 [file molecules-21-00779-s001.pdf]

Molecules **2016**, *21*, 779; doi:10.3390/molecules21060779

Supplementary Materials: Chemical Structure and Immunomodulating Activities of an α-Glucan Purified from *Lobelia chinensis* Lour

Xiao-Jun Li, Wan-Rong Bao, Chung-Hang Leung, Dik-Lung Ma, Ge Zhang, Ai-Ping Lu, Shun-Chun Wang and Quan-Bin Han

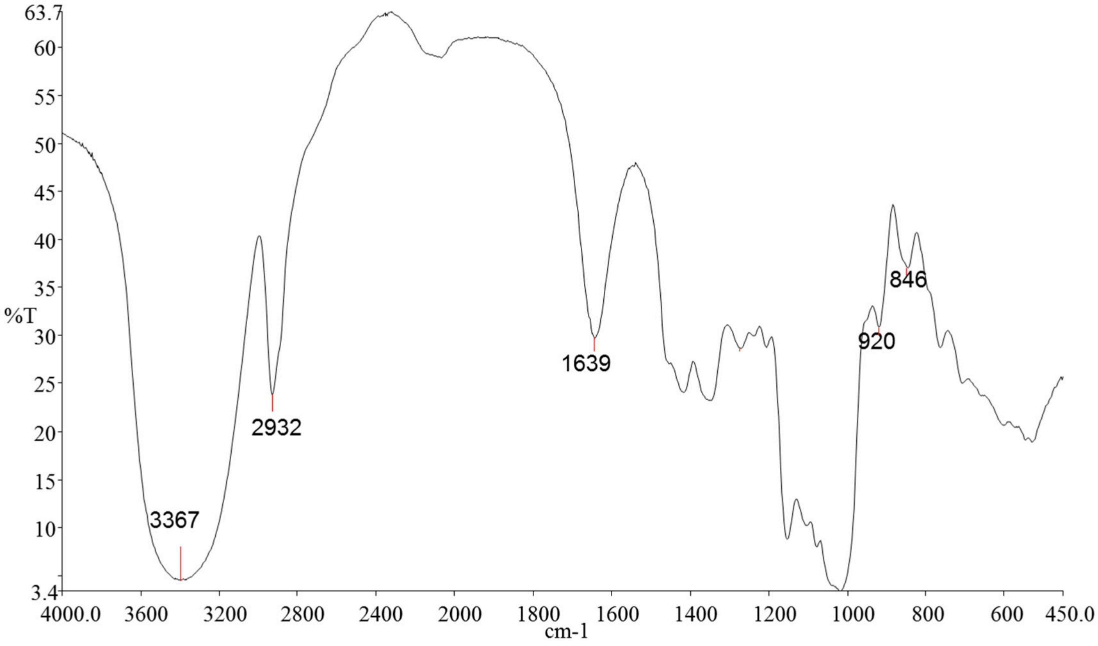

Figure S1. The IR spectrum of BP1.
